# Increased Precipitation and Nitrogen Alter Shrub Architecture in a Desert Shrubland: Implications for Primary Production

**DOI:** 10.3389/fpls.2016.01908

**Published:** 2016-12-20

**Authors:** Weiwei She, Yuqing Zhang, Shugao Qin, Bin Wu, Yuxuan Bai

**Affiliations:** ^1^Yanchi Research Station, School of Soil and Water Conservation, Beijing Forestry UniversityBeijing, China; ^2^Key Laboratory of State Forestry Administration on Soil and Water Conservation, Beijing Forestry UniversityBeijing, China; ^3^Engineering Research Center of Forestry Ecological Engineering, Ministry of Education, Beijing Forestry UniversityBeijing, China

**Keywords:** *Artemisia ordosica*, dryland, architectural traits, twig size and number, trade-off, nitrogen deposition, global environmental changes

## Abstract

Shrublands are one of the major types of ecosystems in the desert regions of northern China, which is expected to be substantially more sensitive to global environmental changes, such as widespread nitrogen enrichment and precipitation changes, than other ecosystem types. However, the interactive effects of nitrogen and precipitation on them remain poorly understood. We conducted a fully factorial field experiment simulating three levels of precipitation (ambient, +20%, +40%) and with two levels of nitrogen deposition (ambient, 60 kg N ha^-1^ yr^-1^) in a desert shrubland in the Mu Us Desert of northern China. We used plant architectural traits (plant cover, volume, twig size and number) as proxies to predict aboveground net primary productivity (ANPP) of the dominant shrub (*Artemisia ordosica* Krasch), and assessed the responses of plant productivity and architectural traits to water and nitrogen addition. We found significant differences in twig size and number of *A. ordosica* under water and nitrogen treatments but not in shrub cover/volume, which suggest that twig size and number of the shrub species were more sensitive to environmental changes. The productivity of the overall community was sensitive to increased precipitation and nitrogen, and shrubs played a more important role than herbaceous plants in driving productivity in this ecosystem. Precipitation- and nitrogen-induced increases in vegetation production were positively associated with increases in twig size and number of the dominant shrub. Water addition enhanced the twig length of *A. ordosica*, while nitrogen addition resulted in increased twig density (the number of twigs per square meter). Water and nitrogen interacted to affect twig length, but not twig number and shrub ANPP. The trade-off, defined as negative covariance between twig size and number, was likely the mechanism underlying the responses of twig length and shrub ANPP to water and nitrogen interactions. Our results highlight the sensitivity of twig size and number as indicators to estimate shrub production and the mechanism underpinning desert shrub ANPP response to global environmental changes.

## Introduction

Global environmental changes project widespread nitrogen enrichment and precipitation changes in the future (Galloway et al., 2008; IPCC, 2013). These changes are expected to profoundly impact the structure and function of terrestrial ecosystems, especially in water- and nutrient-limited drylands (Bobbink et al., 2010; Maestre et al., 2016). Drylands in China are expected to see an increase in both precipitation (IPCC, 2013) and atmospheric nitrogen deposition (Liu et al., 2013) in the coming decades. Shrublands are one of the major types of dryland ecosystems, especially in desert regions of northern China, yet the interactive effects of global change factors remain poorly understood. Therefore, knowledge of how global changes will affect dryland shrublands in the future is crucial for their adaptive management (Nicholson, 2011).

Plant functional traits are the attributes (morphological, physiological and phenological) that represent plant ecological strategies (growth, reproduction and survival) (Violle et al., 2007; Gotmark et al., 2016). These attributes are closely related to ecosystem functions and have been widely used as predictors of plant responses to environmental changes (Gornish et al., 2014; Lv et al., 2016). Plant architectural traits (e.g., plant cover, volume, twig size and number) correlate with water-use efficiency (Westoby et al., 2002; Valencia et al., 2015) and are usually used as proxies to evaluate biomass and primary production (Jobbágy and Sala, 2000; Flombaum and Sala, 2007). Plant cover is a good predictor of aboveground biomass in arid systems (Flombaum and Sala, 2007), and thus it can be a suitable proxy for aboveground net primary production (ANPP) in herb-dominated systems. However, shrub aboveground biomass consists of current-year branch biomass and old branch biomass which accounts for a large fraction of the total plant biomass (Castro and Freitas, 2009). Shrub current-year branch biomass is most commonly used as a proxy for ANPP (Jobbágy and Sala, 2000) since plant cover/volume is more related to vegetation biomass rather than ANPP in shrub-dominated ecosystems. To avoid clipping effects in multiyear manipulation experiments, non-destructive methods that use plant cover/volume as a proxy for ANPP are commonly used to estimate ecosystem ANPP (Gherardi and Sala, 2015). Many field studies showed that herbaceous cover/production significantly increased with water and/or nitrogen addition (Hall et al., 2011; Peters et al., 2012), while shrub cover/volume showed limited (Peters et al., 2012; Vourlitis, 2012; Baez et al., 2013) or non-significant (Snyder et al., 2004; Reichmann et al., 2013) responses to water and nitrogen addition. However, few studies have tested the interactive effects of water and nitrogen on shrub cover/volume. Results from previous studies that used plant cover/volume as a proxy to estimate ANPP suggest that precipitation- or nitrogen-induced changes in dryland ANPP are primarily associated with herbaceous plants (Peters et al., 2012; Reichmann et al., 2013).

Alternatively, twig size and number are also useful indicators of shrub production. In other words, twig size and number are good surrogates of twig biomass, a good proxy of shrub ANPP (Jobbágy and Sala, 2000). Results from many studies that tested enhanced precipitation or nutrient effects on the growth of shrub twigs/branches were inconsistent, and the interactive effects of water and nitrogen addition on twig size are seriously scarce. Many field experiments found that shrub twig/branch elongation was unresponsive (Lajtha and Whitford, 1989; Yahdjian and Sala, 2006) or responded positively (Fisher et al., 1988; Sponseller et al., 2012; Zhang et al., 2015) to increased precipitation. Similarly, nutrient enrichment resulted in either non-significant effects (Lajtha and Whitford, 1989; Hall et al., 2011) or positive effects (Fisher et al., 1988; Drenovsky and Richards, 2005) on shrub twig/branch elongation. Twig elongation is an important aspect of shrub growth but not a complete surrogate of ANPP (Sponseller et al., 2012). However, few studies examined twig number response to precipitation and nitrogen addition. Compared to using architectural traits to predict shrub production indirectly, Jobbágy and Sala (2000) harvested twig biomass and found that shrub ANPP was associated with precipitation in Patagonian steppe.

In this study, we explored two main hypotheses regarding the shrub architectural traits and ANPP responses to precipitation and nitrogen enrichment. First, we hypothesized that shrub cover/volume would have limited sensitivity in response to environmental changes resulting in a failure to capture the variation in shrub production. Shrub cover/volume is more related to plant biomass and likely underestimates the small biomass increases annually due to the large fraction of old branch systems in the plant architecture. Second, we hypothesized that twig size and number would be linked to the response of shrub production to enhanced precipitation and nitrogen. We expected that increased precipitation would enhance the length and number of shrub twigs, and nitrogen enrichment affect twig length and number depending on water availability. The twig growth of desert shrubs is expected to be primarily nitrogen limited when soil moisture is sufficient (Hall et al., 2011). To test these hypotheses, we conducted a field experiment with increased precipitation and nitrogen in a desert shrubland in the Mu Us Desert of northern China. We assessed the effects of water and nitrogen addition on ANPP and plant cover, volume, twig size and number in *Artemisia ordosica* Krasch, the dominant shrub in the study area.

## Materials and Methods

### Study Area and Species

The field experiment was conducted at Yanchi Research Station (37°04′–38°10′N, 106°30′–107°41′E, 1530 m a.s.l), which is situated on the southwestern edge of the Mu Us Desert, Ningxia, China. There is little variation in the topographic relief here with ∼50 m difference in elevation from the lowland to upland sites. The climate in this area is a mid-temperate and semiarid continental monsoon climate with a mean annual temperature of 8.1°C. The average annual precipitation is 284.8 mm (1955–2013), of which 83.3% falls from May to September (Supplementary Table S1). Soil type is quartisamment according to the US Soil Taxonomy (Gao et al., 2014). The landscape is characterized by patchy vegetation and bare soils with biological soil crusts. The dominant plant species in this region is *A. ordosica*, with sparse shrubs *Hedysarum mongolicum, Salix psammophila, Caragana korshinskii*, and grass *Agropyron cristatum*. Grazing in this area has been prohibited since the late 1990s and vegetation has been recovered for over a decade (Jia et al., 2014).

*Artemisia ordosica* is a xeric, deciduous, multi-stemmed, dwarf shrub with plumose, linearly lobate leaves and a height ranging from 50 to 100 cm. Its lateral roots are primarily distributed to depths of 0–30 cm, and its tap roots can reach 1–3 m deep. The branch system consists of old branches (brown) and current-year twigs (CYT, purple, Supplementary Figure S1A). The CYT sprout from old branches and stems and consist of vegetative and reproductive twigs. Most vegetative twigs survive the winter and bear new vegetative or reproductive twigs the following spring, while reproductive twigs die in winter (Li et al., 2011).

### Experimental Design

The precipitation and nitrogen manipulation experiment was established at a lowland site in October 2014 using a two-factor experiment. There were three precipitation levels (W0: ambient, W20: ambient + 20%, and W40: ambient + 40%) and two nitrogen levels (N0: no additional nitrogen and N60: 60 kg N ha^-1^ yr^-1^) resulting in a total of six treatments (including all permutations of the two factors), each with four replications. Plots (each 5 m × 5 m with a 1 m wide buffer zone) were laid out in a randomized block design (four blocks with six treatment plots within each block).

The magnitude of precipitation enhancement was based on long-term mean annual precipitation (1955-2013: 284.8 mm). Specifically, the W20 and W40 treatments received approximately 56 and 112 mm supplementary precipitation, respectively. According to the amount and distribution of long-term mean monthly precipitation in this area (Supplementary Table S1), water was added with a sprinkler irrigation system nine times in equal amount during the growing season (May-September) with three times occurring in July and August and one time each in May, June, and September (Supplementary Figure S1B). In order to not alter the patterns of current year precipitation, water was added following a natural rainfall event. Detailed information on the dates of water addition are shown in Supplementary Figure S2. In this study, we defined annual precipitation as the water-year precipitation received between October 1 and the following September 30. Ambient water-year precipitation was 288 mm in 2014-2015.

Ammonium nitrate solution was applied evenly over the plots using a sprayer as five equal applications at the beginning of each month during the growing season. During each application, 85.71 g NH_4_NO_3_ (an analytical reagent) was weighted and dissolved in 10 L tap water (equivalent to concentration of 0.21 mol N L^-1^). The nitrogen control plots received the same amount of water, equivalent to a 2 mm precipitation, without added nitrogen. Background nitrogen deposition in study site was about 12 kg N ha^-1^ yr^-1^ according to our monitoring data (She, unpublished data). The mean nitrogen deposition rate in northern China was 56.2 kg N ha^-1^ yr^-1^ (Xu et al., 2015). Nitrogen addition plots received nitrogen at rate of 60 kg N ha^-1^ yr^-1^, an amount selected to simulate areas that have received high deposition.

### Field Sampling and Measurements

We classified all plants into two functional groups according to their life forms, including shrubs and herbaceous plants (see Supplementary Table S2). In the middle of September 2015, shrubs were counted and measured [height (*H*), maximum crown width (*CW_1_*), and minimum crown width (*CW_2_*)] in each 5 m × 5 m plot. With these measurements, individual cover (*C*) and volume (*V*) of the shrubs were calculated as *C* = π ×*CW_1_*/2 ×*CW_2_*/2 and *V* = *C* ×*H*, respectively. Shrub cover and volume were calculated as the sum of the individual values in each plot and expressed as m^2^ m^-2^ and m^3^ m^-2^, respectively. Herbaceous plants were harvested at the soil surface using a 1 m × 1 m quadrat randomly placed in each plot. All plants were sorted by species and oven-dried at 75°C for 48 h and weighed. We used biomass measurements at the peak growth period as estimation of herbaceous ANPP (Sala and Austin, 2000).

For the purpose of this study, we used two methods for shrub (*A. ordosica*) ANPP estimation. One was the traditional method in which plant volume was used as a proxy for individual shrub ANPP, and the other was an improved, non-destructive method based on twig size and number. For the traditional method, we used an allometric equation (*ANPP_traditional_* = 193.433 ×*V*^0.906^, *R*^2^ = 0.79, *n* = 31, *P* < 0.01) developed in a similar habitat adjacent to our experimental field (She et al., 2015). Shrub ANPP (g m^-2^) was the sum of the individual values at the plot level. For the improved method, we considered the dry mass of CYT (twig biomass) as a proxy for shrub ANPP, which could be easily recognized by their color and structure (Jobbágy and Sala, 2000). This estimation method may slightly underestimate shrub ANPP due to not accounting for the growth of old branches, but it has been successful for shrub ANPP prediction in the Patagonian steppe (Jobbágy and Sala, 2000) and Mediterranean rangeland (Castro and Freitas, 2009). We estimated twig biomass by measuring the average size and number of twigs. Specifically, we established allometric relationships between twig length and biomass to predict single twig biomass, and then the total twig plant biomass was estimated by the number of twigs and mean twig length. In this study, we randomly sampled ∼20 vegetative twigs and ∼10 reproductive twigs of *A. ordosica* in each plot. These twigs were measured and their dry mass was obtained after oven-drying at 75°C for 48 h. We found that water and nitrogen treatments had no effects on the allometric relationships between twig length and biomass by using the standardized major axis (SMA) regression method [for vegetative twigs: Likelihood ratio statistic (*LRS*) = 5.666, *P* = 0.340, Supplementary Figure S3A; for reproductive twigs: *LRS* = 7.413, *P* = 0.192, Supplementary Figure S3B]. Thus, we combined data from all treatments to develop the allometric equations for estimating twig biomass using twig length as an independent variable (see Supplementary Figures S3C,D).

In the middle of September 2015, vegetative and reproductive twigs were counted manually within permanent 1 m × 1 m quadrats located in an *A. ordosica* patch representative of the plot. Then, we randomly measured the length of ∼20 vegetative twigs and ∼10 reproductive twigs. Using the developed allometric equations, the average biomass of vegetative and reproductive twigs in the plant patch was estimated. Combining data of the average biomass and number of the two types of twigs, we obtained shrub ANPP (g m^-2^) in the plant patch. We measured shrub coverage (%) with three parallel lines in each plot, each of 5 m length. Shrub ANPP (g m^-2^) in each plot was the product of the shrub ANPP in the plant patch and the shrub coverage in the plot.

Soil moisture and temperature were monitored in all treatments in one single block at 10 cm below the soil surface using ECH_2_O-5TE sensors (Decagon Devices, USA). Probes were calibrated for soils at the study site following the oven drying method (calibration *R*^2^ = 0.94, *n* = 21, *P* < 0.01).

### Statistical Analyses

We used ANOVAs (type I effect computations) to test the main and interactive effects of nitrogen and water addition on shrub cover and volume, shrub ANPP estimated from the traditional and improved methods, the density (twigs m^-2^) and length of shrub CYT, herbaceous and community ANPP. Community ANPP was the sum of shrub ANPP estimated from the improved method and herbaceous ANPP. One-way ANOVAs with Duncan’s multiple range tests were performed to compare the watering effects on CYT length at each nitrogen addition rate and the nitrogen effects at each water-treatment level. We used linear models to test the relationship between twig size (twig length, cm) and number (twigs m^-2^) of *A. ordosica*. Standardized major axis (SMA) regression was used to determine the slopes and intercepts of allometric relationships between twig lengths and biomasses, and all values were ln-transformed prior to analysis. We compared the difference in allometric slopes among different treatments. Linear models for predicting the biomass of the two type twigs were developed with twig length as an independent variable using untransformed data of vegetative twigs and ln-transformed data of reproductive twigs. For all analyses, statistical significance was determined at a level of *P* ≤ 0.05. The data of herbaceous ANPP were ln-transformed to meet the assumption of normality. All analyses and figures were performed with R version 3.3.1 (R Core Team, 2016). We used the package SMATR 3 (Warton et al., 2012) for SMA regressions and ggplot2 (Wickham, 2009) for graphs.

## Results

**FIGURE 1:**
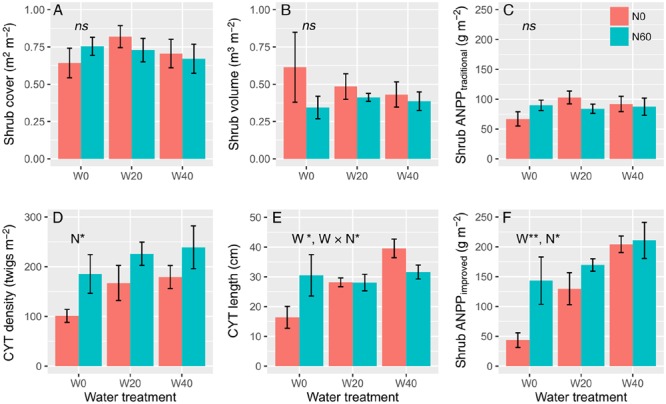
**Effects of water and nitrogen addition on shrub cover (A)**, shrub volume **(B)**, shrub ANPP estimated from the traditional **(C)** and improved methods **(F)**, and twig density **(D)** and length **(E)** of *A. ordosica* (^∗^*P* < 0.05, ^∗∗^*P* < 0.01; *ns*, not significant).

Water and nitrogen addition had non-significant effects on shrub cover, volume, or ANPP estimated from the traditional method, but had remarkable effects on the density and length of CYT and shrub ANPP estimated from the improved method (**Figure 1**, Supplementary Table S3). *Artemisia ordosica* CYT density significantly increased only in plots with added nitrogen, from 149.20 ± 16.83 (N0 treatments) to 216.87 ± 20.08 twigs m^-2^ (N60), a dramatic increase rate of 45.36% on average (*F* = 7.616, *P* = 0.015, **Figure 1D**). Water addition significantly enhanced the CYT length of *A. ordosica* (*F* = 4.933, *P* = 0.023) and nitrogen had no impacts on its length (*F* = 0.402, *P* = 0.535). Water and nitrogen interacted to affect the CYT length (*F* = 4.117, *P* = 0.038), to the effect that water-induced changes in CYT length were more significant in the unfertilized (Duncan’s test, *P* < 0.05) than in fertilized plots (Duncan’s test, *P* > 0.05). Unexpectedly, nitrogen-induced increases in the CYT length were found in the ambient plots (W0, Duncan’s test, *P* < 0.05), but not in watering plots (W20 and W40, Duncan’s test, *P* > 0.05). Both water (*F* = 9.764, *P* = 0.002) and nitrogen (*F* = 5.323, *P* = 0.036) treatments increased shrub ANPP estimated from the improved method, but no interactive effects were found (*F* = 1.672, *P* = 0.221). Watering enhanced shrub ANPP from 93.50 ± 26.92 (W0) to 149.67 ± 15.35 (W20) and 207.60 ± 15.41 g m^-2^ (W40), a remarkable increase rate of 60.07 and 122.03% on average, respectively. Nitrogen-induced increases in shrub ANPP in the fertilized plots (N60, 174.58 ± 17.50 g m^-2^) were 38.62% higher than that in unfertilized plots (N0, 125.94 ± 22.11 g m^-2^) on average.

**FIGURE 2:**
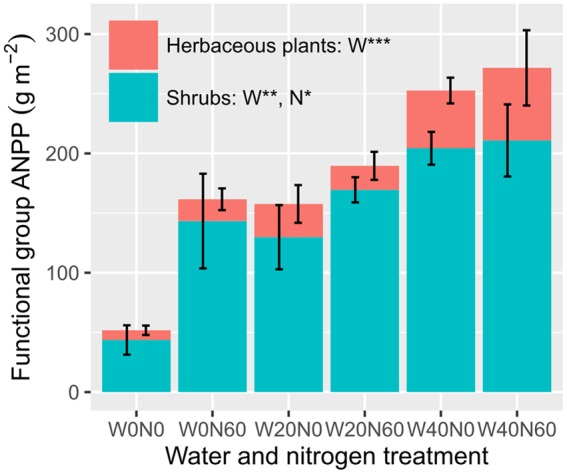
**Effects of water and nitrogen addition on functional group ANPP (^∗^*P* < 0.05, ^∗∗^*P* < 0.01, ^∗∗∗^*P* < 0.001; *ns*, not significant)**.

**FIGURE 3:**
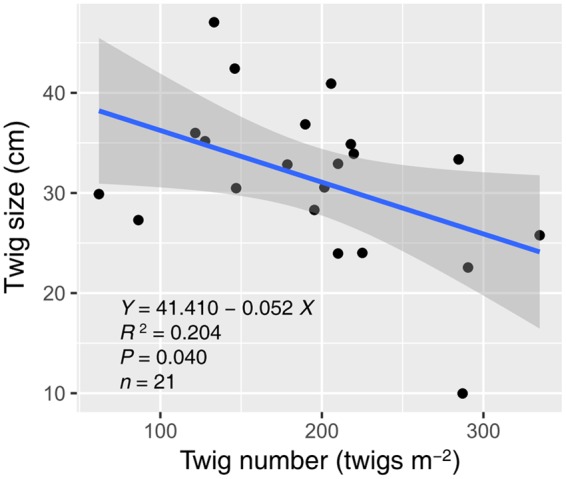
**Relationship between twig size and number of *A. ordosica*.** The gray region indicates the 95% confidence interval around the regression. Three abnormal observations were removed prior to regression analysis.

Water and nitrogen enrichment had different effects on functional group ANPP (**Figure 2**, Supplementary Table S3). Water addition increased the ANPP of herbaceous plants (*F* = 12.728, *P* < 0.001), shrubs (*F* = 9.764, *P* = 0.002), and communities (*F* = 13.320, *P* < 0.001). Nitrogen enrichment resulted in an increase in shrub (*F* = 5.323, *P* = 0.036) and community ANPP (*F* = 4.716, *P* = 0.046), but did not affect herbaceous plants (*F* = 0.056, *P* = 0.816). No significant interactive effects of water and nitrogen addition were found in any case (Supplementary Table S3). The average herbaceous ANPP in W20 (23.96 ± 9.23 g m^-2^) and W40 (54.59 ± 15.61 g m^-2^) treatments were 81.65 and 313.87% higher than that in the ambient treatments (W0, 13.19 ± 4.97 g m^-2^), respectively. Shrub ANPP accounted for 83.7% of community productivity in all treatments on average. Moreover, we found twig size was significantly negatively correlated with twig number, suggesting the existence of a trade-off between these two traits (*R^2^* = 0.204, *P* = 0.040, **Figure 3**).

## Discussion

Our results support the hypothesis that plant cover/volume would be not a sensitive indicator of shrub ANPP under environmental changes. We found significant differences in twig size and number of *A. ordosica* under water and nitrogen addition but not in plant cover/volume, which suggest that twig size and number were more sensitive to environmental changes and using plant cover/volume as a predictor for shrub performance was likely to underestimate the effects of environmental changes on desert shrub ANPP as in previous studies. Since shrub canopy architecture accumulates over the course of several years, shrub cover/volume is more related to plant biomass rather than ANPP. Shrub cover/volume is expected to capture the variation of plant ANPP when plant canopy experiences significant growth in wetter years or with sufficient resource availability (Vourlitis, 2012). However, it is not to say that shrub cover/volume can not be used as an indicator for individual performance. The suitable plant traits as the predictors for individual performance will depend on the characteristics of the ecosystem and the objectives of the study. For example, shrub cover responses dramatically to the disturbance of grazing and fire, suggesting that it is suitable as an indicator for shrub performance in grazing and postfire systems (Eldridge et al., 2013; Kimball et al., 2014). When in an undisturbed ecosystem, shrub cover may not be a good predictor of individual performance due to its weak response to environmental changes. Twig size and number may be more suitable indicators of shrub performance in response to environmental changes. However, they have two disadvantages. Firstly, they may perform well in dwarf shrubs such as *A. ordosica*, but not in tall shrubs with large branching architecture. Dwarf shrubs accumulate most of their annual production through growth of CYTs, but tall shrubs through increases in branch or stem diameter. Secondly, twig size and number are not easy-to-measure traits compared to plant cover.

Our results are also in line with the second hypothesis that enhanced precipitation and nitrogen would increase desert shrubland productivity through altering shrub twig size and number. Our results support previous findings that water addition promotes shrub branch elongation (Fisher et al., 1988; Sponseller et al., 2012; Zhang et al., 2015). Unexpectedly, it was nitrogen addition that had significant effects on twig number, instead of water addition (**Figure 1D**). Nitrogen-induced increases in shrub twig number were likely due to an increase in soil nitrogen availability, which could activate sprouting from buds through stimulating cytokinin production from root (Takei et al., 2001; Tomlinson and O’Connor, 2004). Many studies in grasslands also found that nitrogen addition results in increased tiller density (Tomlinson and O’Connor, 2004; Dalgleish et al., 2008). Previous studies suggested that nitrogen primarily limits the branch elongation of desert shrubs when water limitation is alleviated (Fisher et al., 1988; Hall et al., 2011). However, the data from our study showed that water addition suppressed nitrogen-induced elongation in shrub twigs (**Figure 1E**). The possible interpretation was that the nitrogen-treatment effects on twig elongation might be diluted following excessive water addition. The mechanism of interactive effects of water and nitrogen on twig length was more likely due to the trade-off, defined as negative covariance between twig size and number (**Figure 3**). Trade-off represents the balancing of energy allocation to different traits based on an organism’s limited resources when a beneficial change in one trait is linked to a detrimental change in another (Stearns, 1989). Previous studies showed that twig size-number trade-off correlates tightly with the biomass allocation strategies of woody species under varying environmental conditions (Sun et al., 2010; Xu et al., 2012). In our study, because of increased twig number with nitrogen addition, the twig size-number trade-off could limit precipitation-induced increases in twig length, which explained the interactive effects of water and nitrogen on twig length. This trade-off also constrained shrub ANPP increases under high resource availability by limiting twig number and length from attaining simultaneously high growth. They explained why no interactive effects of water and nitrogen on shrub ANPP were found in our study.

The data collected in this study show that different plant functional groups respond in different ways to environmental changes. Water and nitrogen addition increased shrub ANPP via enhancing the twig length and number of *A. ordosica*. The increased herbaceous production with water addition was more related to plant cover (data not shown). Since the shrubs occupied a large fraction of community ANPP, their role was more important than herbaceous plants in controlling primary production in this ecosystem. Our results agree with the findings that shrub ANPP correlates tightly with precipitation (Jobbágy and Sala, 2000; Sponseller et al., 2012), but are not in line with that precipitation- or nitrogen-induced changes in herbaceous productivity accounts for the majority of total ANPP variation in arid shrublands (Hall et al., 2011; Peters et al., 2012). The inconsistent results from different studies were likely due to the different methods for estimation of shrub ANPP. Our findings are the first results and based on only one site. Further study using different methods for estimating shrub ANPP is needed to assess the contribution of shrubs, relative to that of herbaceous plants to dryland productivity in response to global environmental changes.

## Conclusions and Implications

Although, in our short-term experiment, we found that *A. ordosica*-dominated shrubland productivity was sensitive to increased precipitation and nitrogen. Shrubs were more crucial than herbaceous plants in driving ecosystem productivity because of their dominant role in the community. Precipitation- and nitrogen-induced increases in community productivity were positively associated with increases in twig size and number of the dominant shrub. Twig size-number trade-off was likely the mechanism regulating the effects of water and nitrogen addition on *A. ordosica* ANPP. Our results highlight the sensitivity of twig size and number as indicators to estimate shrub ANPP under environmental changes. We firstly found that twig size and number played a crucial role in mediating *A. ordosica* ANPP response to water and nitrogen addition. Our research contributes to understand the mechanism underlying desert shrub ANPP response to global environmental changes. However, further research is needed to test the validity of this mechanism in other xeric shrub species in arid systems.

## Author Contributions

WS and YZ designed the experiment; WS and YB carried out the field work; WS analyzed the data; WS and YZ wrote the manuscript; YZ, SQ, and BW assisted with revising the draft manuscript.

## Conflict of Interest Statement

The authors declare that the research was conducted in the absence of any commercial or financial relationships that could be construed as a potential conflict of interest.
