# Supplementary material for: Increased Precipitation and Nitrogen Alter Shrub Architecture in a Desert Shrubland: Implications for Primary Production
Source: Front Plant Sci. 2016 Dec 20;7:1908. doi: 10.3389/fpls.2016.01908 (PMC5167761; doi:10.3389/fpls.2016.01908)
Supplement: Supplementary file 1 [file Data_Sheet_1.DOCX]

Supplementary Material

**Increased precipitation and nitrogen alter shrub architecture in a desert shrubland: implications for primary production**

**Weiwei She, Yuqing Zhang^*^, Shugao Qin, Bin Wu, Yuxuan Bai**

*** Correspondence:** Yuqing Zhang: zhangyqbjfu@gmail.com

**Table S1.** Mean percent annual share of precipitation per month for 1955 to 2013 in the study area

| Month | 10 | 11 | 12 | 1 | 2 | 3 | 4 | 5 | 6 | 7 | 8 | 9 |
| --- | --- | --- | --- | --- | --- | --- | --- | --- | --- | --- | --- | --- |
| Percentage (%) | 6.4 | 1.8 | 0.3 | 0.4 | 0.6 | 2.2 | 5.0 | 9.6 | 11.8 | 21.2 | 25.5 | 15.2 |

**Table S2.** Functional groups and plant species found in experimental plots in the Mu Us Desert

| Functional group | Species |
| --- | --- |
| Shrubs | *Artemisia ordosica* |
| Herbaceous plants (Perennial grasses) | *Leymus secalinus, Pennisetum centrasiaticum, Agropyron cristatum, Stipa glareosa,* |
| Herbaceous plants (Perennial forbs) | *Ixeris chinensis, Heteropappus altaicus, Cynanchum thesioides, Euphorbia esula* |
| Herbaceous plants (Annuals) | *Incarvillea sinensis, Euphorbia humifusa, Corispermum puberulum, Chenopodium aristatum, Bassia dasyphylla, Eragrostis poaeoides, Setaria viridis* |

**Table S3.** Results of ANOVAs of the effects of water addition (W), nitrogen addition (N), and their interaction (W × N) on shrub cover and volume, shrub ANPP estimated from the traditional method (ANPP_traditional_) and the improved method (ANPP_improved_), CYT density and length of *Artemisia ordosica*, and ANPP of functional groups. All results with *P* ≤ 0.05 are in boldface. *F*-test values (*F*) and *P*-values (*P*) are given

| Effect | Shrub cover | | Shrub volume | | Shrub ANPP_traditional_ | | CYT density | |
| --- | --- | --- | --- | --- | --- | --- | --- | --- |
|  | *F* | *P* | *F* | *P* | *F* | *P* | *F* | *P* |
| W | 1.260 | 0.312 | 0.192 | 0.827 | 1.608 | 0.233 | 2.708 | 0.099 |
| N | 0.009 | 0.927 | 1.930 | 0.185 | <0.001 | 0.983 | 7.616 | **0.015** |
| W × N | 1.566 | 0.241 | 0.577 | 0.574 | 2.869 | 0.088 | 0.117 | 0.891 |

continued

| Effect | CYT length | | Shrub ANPP_improved_ | | Herbaceous ANPP | | Community ANPP | |
| --- | --- | --- | --- | --- | --- | --- | --- | --- |
|  | *F* | *P* | *F* | *P* | *F* | *P* | *F* | *P* |
| W | 4.933 | **0.023** | 9.764 | **0.002** | 12.728 | **<0.001** | 13.320 | **<0.001** |
| N | 0.402 | 0.535 | 5.323 | **0.036** | 0.056 | 0.816 | 4.716 | **0.046** |
| W × N | 4.117 | **0.038** | 1.672 | 0.221 | 2.243 | 0.141 | 1.321 | 0.296 |


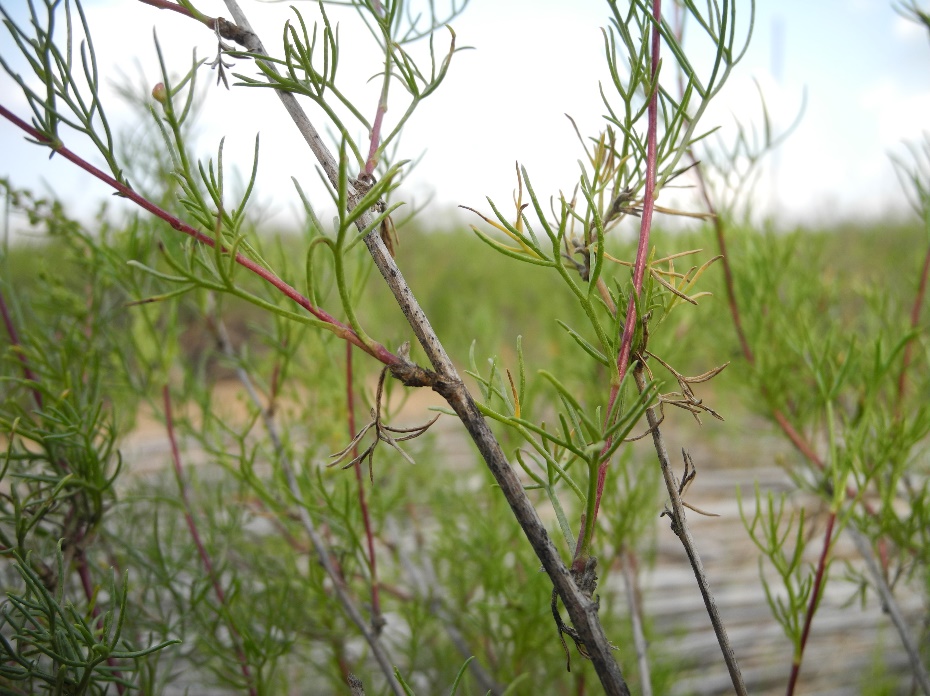

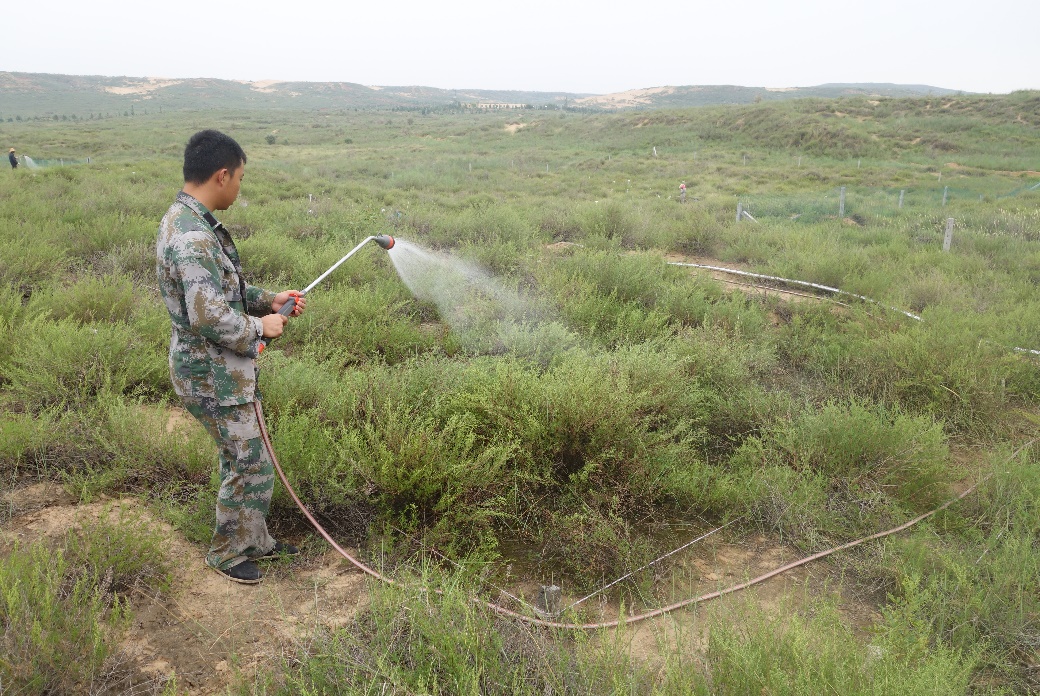


A

B

**Figure S1.** The branch architecture of *Artemisia ordosica* (A), with brown older branchs and purple current-year twigs and photograph of a experimental plot (B) (we were watering with a sprinkler irrigation system).


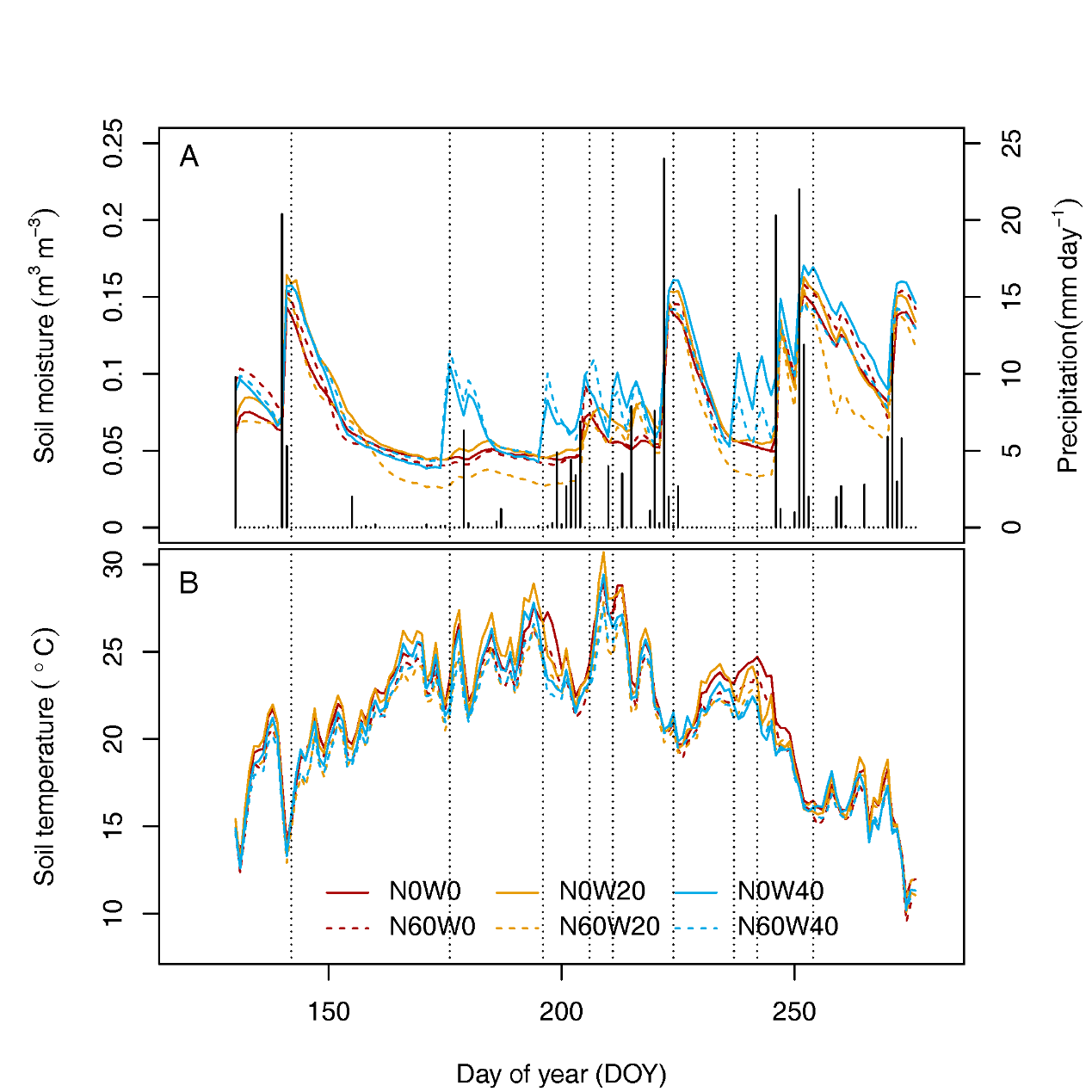


**Figure S2.** Temporal trends of precipitation and soil moisture (A) and temperature (B) at 10 cm soil depth in the six water and nitrogen addition treatment plots of one block in the 2015 growing season. The vertical dashed lines represent the dates of irrigation. The solid lines indicate precipitation events. Water addition successfully increased soil moisture but did not alter its temporal patterns. N0W0, no additional nitrogen and ambient precipitation; N0W20, no additional nitrogen and ambient+20% precipitation; N0W40, no additional nitrogen and ambient+40% precipitation; N60W0, 60 kg N ha^-1^ yr^-1^ and ambient precipitation; N60W20, 60 kg N ha^-1^ yr^-1^ and ambient+20% precipitation; N60W40, 60 kg N ha^-1^ yr^-1^ and ambient+40% precipitation.


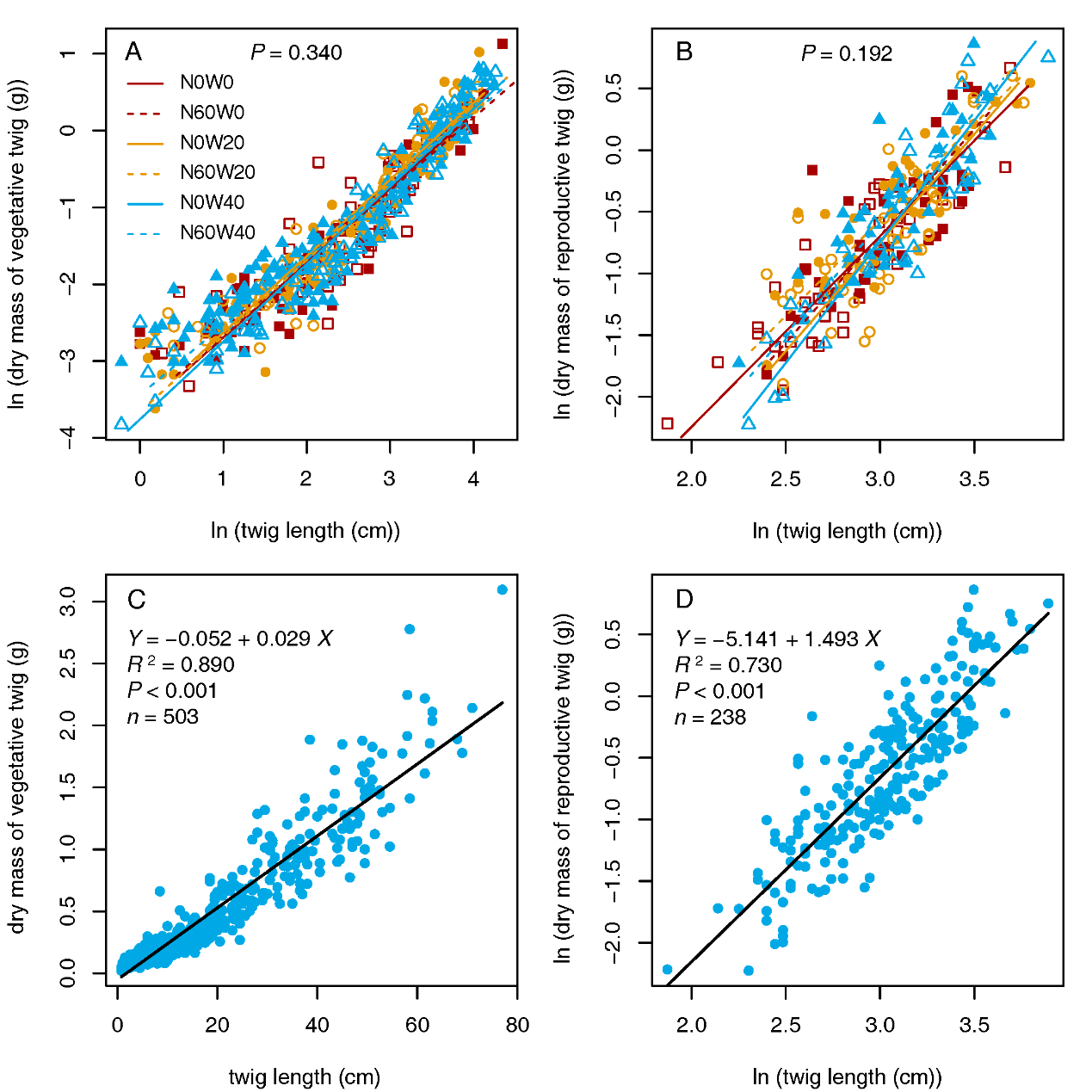


**Figure S3.** Allometric relationships between *Artemisia ordosica* twig dry mass and length. Mass-length relationships of vegetative twig (A) and reproductive twigs (B) of *Artemisia ordosica* at various water and nitrogen addition levels. General allometric equations for estimating the dry mass of vegetative twigs (C) and reproductive twigs (D) over all experimental treatments using twig length as an independent variable. N0W0, no additional nitrogen and ambient precipitation; N0W20, no additional nitrogen and ambient+20% precipitation; N0W40, no additional nitrogen and ambient+40% precipitation; N60W0, 60 kg N ha^-1^ yr^-1^ and ambient precipitation; N60W20, 60 kg N ha^-1^ yr^-1^ and ambient+20% precipitation; N60W40, 60 kg N ha^-1^ yr^-1^ and ambient+40% precipitation.
